# Deficient AMPK activity contributes to hyperexcitability in peripheral nociceptive sensory neurons and thermal hyperalgesia in lupus mice

**DOI:** 10.1371/journal.pone.0288356

**Published:** 2023-07-13

**Authors:** Viacheslav Viatchenko-Karpinski, Lingwei Kong, Han-Rong Weng

**Affiliations:** 1 Department of Biomedical Sciences, Mercer University School of Medicine, Macon, GA, United States of America; 2 Department of Basic Sciences, California Northstate University College of Medicine, Elk Grove, CA, United States of America; North Carolina State College of Veterinary Medicine, UNITED STATES

## Abstract

Patients with systemic lupus erythematosus (SLE) often suffer from chronic pain. Little is known about the peripheral mechanisms underlying the genesis of chronic pain induced by SLE. The aim of this study was to investigate whether and how membrane properties in nociceptive neurons in the dorsal root ganglions (DRGs) are altered by SLE. We found elevation of resting membrane potentials, smaller capacitances, lower action potential thresholds and rheobases in nociceptive neurons in the DRGs from MRL/lpr mice (an SLE mouse model) with thermal hyperalgesia. DRGs from MRL/lpr mice had increased protein expressions in TNFα, IL-1β, and phosphorylated ERK but suppressed AMPK activity, and no changes in sodium channel 1.7 protein expression. We showed that intraplantar injection of Compound C (an AMPK inhibitor) induced thermal hyperalgesia in normal mice while intraplantar injection of AICAR (an AMPK activator) reduced thermal hyperalgesia in MRL/Lpr mice. Upon inhibition of AMPK membrane properties in nociceptive neurons from normal control mice could be rapidly switched to those found in SLE mice with thermal hyperalgesia. Our study indicates that increased excitability in peripheral nociceptive sensory neurons contributes to the genesis of thermal hyperalgesia in mice with SLE, and AMPK regulates membrane properties in nociceptive sensory neurons as well as thermal hyperalgesia in mice with SLE. Our study provides a basis for targeting signaling pathways regulating membrane properties of peripheral nociceptive neurons as a means for conquering chronic pain caused by SLE.

## Introduction

Systemic lupus erythematosus (SLE) is a chronic autoimmune disease in which the immune system mistakenly produces antibodies against the body’s own tissues, causing damage in diverse tissues in the body, and various clinical manifestations. The majority of SLE patients live with chronic pain despite the reduction in mortality associated with SLE in recent years [1]. Management of chronic pain in patients with SLE remains a clinical challenge due to the limited efficacy and safety profile of current analgesics [1]. Understanding cellular and molecular mechanisms underlying the genesis of chronic pain induced by SLE is a prerequisite for the development of novel strategies to combat chronic pain in patients with SLE.

MRL/Lpr mice are widely used as animal models for the study of SLE. MRL/Lpr mice are homozygous for a spontaneous mutation in the Fas gene, that develop immune dysregulation (production of anti–double-stranded (ds)DNA antibodies) and damages in multiple organs (like kidney and joint) similar to those in SLE patients [2]. Recently, we have reported that MRL/lpr mice spontaneously develop chronic pain [3, 4]. MRL/lpr mice with chronic pain have spinal central sensitization. This was evidenced by enhanced glutamatergic synaptic activities at the first synapse between the primary sensory neurons and the second-order sensory neurons in the spinal dorsal horn, and suppression of spinal glial glutamate transporter activity [3, 4]. However, it remains unknown whether changes in peripheral sensory neurons are implicated in the genesis of chronic pain caused by SLE.

Primary sensory neurons located in the dorsal root ganglion (DRG) give rise to their peripheral processes to innervate the skin, muscles, joints, and visceral organs [5]. DRG sensory neurons are generally grouped into nociceptive neurons and non-nociceptive neurons. Nociceptive neurons are small neurons that give rise to thinly myelinated (Aδ) and unmyelinated (C) fibers [6]. Under normal conditions, in response to peripheral nociceptive stimulation, nociceptive receptors (nociceptors) at the nerve terminals open their ion channels, resulting in an influx of cation ions into the neurons and membrane potential depolarization, a process called nociceptive sensory transduction. Once such depolarization reaches the activation threshold of an action potential, an action potential is generated. Action potentials then propagate along the sensory fibers and transmit the nociceptive signal to the CNS. Neuronal membrane electrophysiological properties are key determinants for the generation and propagation of action potentials in sensory neurons. Previous studies have demonstrated that altered functionality in peripheral nociceptive neurons contributes importantly to the genesis of chronic pain. Abnormal membrane electrophysiological properties in DRG neurons have been reported in animals with pathological pain caused by nerve injury [7, 8], inflammation [9], and bone cancer [10]. Currently, little is known about the membrane electrophysiological features and signaling molecules regulating these features in the primary sensory neurons in mice with chronic pain caused by SLE.

Adenosine monophosphate-activated protein kinase (AMPK) is a key kinase that regulates cellular energy homeostasis [11]. AMPK is activated upon an increase of intracellular AMP/ATP ratio following ATP consumption. AMPK activation leads to a reduction in ATP consumption and an increase in ATP production and restoring of AMP/ATP ratio [11]. Previous studies have shown that AMPK in the dorsal ganglion sensory neurons and the spinal dorsal horn regulate neuropathic pain [12–14], postoperative pain [15], and inflammatory pain induced by complete Freund’s adjuvant (CFA) [16] in rodents. It was shown that function of transient receptor potential ankyrin 1 (TRPA1) in nociceptive sensory neurons in rats with diabetic neuropathic pain is negatively regulated by AMPK [17]. Currently, our understanding about the role of AMPK in the regulation of nociceptive sensory neuronal activity in animals with chronic pain induced by SLE remains unknown.

In this study, we characterized membrane electrophysiological properties in the nociceptive sensory neurons in the DRG in MRL/lpr mice with chronic pain, and defined the role of AMPK in the regulation of the electrophysiological properties and chronic pain induced by SLE.

## Methods and materials

### Animals

Adult male MRL/MpJ^lpr^ (MRL/Lpr) and MRL/Mpj (control) mice were purchased from Jackson Laboratories (Bar Harbor, ME, USA). Animals were housed 3 to 5 per cage in isolated rooms with a 12-hour light and dark cycle (8:00 to 20:00). All experiments were approved by Institutional Animal Care and Use Committee at Mercer University, and were fully compliant with the National Institutes of Health Guidelines for the Use and Care of Laboratory Animals.

### Behavioral tests and drug administration

Behavioral tests were performed to assess thermal sensitivity on the hind paw [3, 4]. Briefly, mice were placed on a glass surface at 30°C under a Plexiglass cage (12 × 20 × 15 cm) and allowed to acclimate for 90 min. A radiant heat beam was directed to the mid-plantar surface of a hind paw to evoke a withdrawal response. The time between the stimulus onset and the paw withdrawal response, i.e., the paw withdrawal latency, was recorded. A cutoff time of 20 seconds was set to prevent damage to the skin. The hind paw was stimulated 3 times with an interval of about 5 mins. Three measured latencies for each paw were averaged.

The AMPK activator (5-Aminoimidazole-4-carboxamide ribonucleotide, AICAR) and the AMPK inhibitor (Compound C) were respectively prepared in saline. MRL/lpr mice were randomly assigned into two groups: AICAR group receiving AICAR treatment, the control group receiving vehicle (saline) treatment. Similarly, control MRL mice were grouped into a Compound C treatment group and a control group receiving vehicle treatment. The tested reagent or saline (in a volume of 25 μl) was injected subcutaneously (s.c.) into the mid-plantar hind paw (5 mm distal from the heel) using a 30-G needle attached to a microsyringe as described previously [18, 19]. The experimenter conducting behavioral tests was blind to types of the mice and the treatments given to the animals.

### Electrophysiology

Patch-clamp recording protocols described previously [20, 21] were used to perform recordings from neurons in the dorsal root ganglion (DRG). Briefly, 16-week-old mice were deeply anesthetized via isoflurane inhalation. Surgery was performed to dissect and harvest DRGs (L4-L5) with their attached nerve bundles. After harvesting, the ganglions were stored in a bath solution on ice and oxygenated with a mixture of 95% O_2_ and 5% CO_2_. The bath solution contained (in mM): NaCl (117.0), KCl (3.5), CaCl_2_ (2.5), MgCl_2_ (1.2), NaH_2_PO_4_ (1.2), NaHCO_3_ (25.0), and Glucose (11.0). Osmolality was adjusted by sucrose to 320 mOsm. Before recording, connective tissues on the surface of DRG were carefully removed with fine forceps. A DRG with their nerve bundles was placed into recording chamber, filled with bath solution, and affixed by a tissue anchor. The DRG was then exposed to 0.05% of Collagenase A and 0.05% Dispase II enzymes for 6 min, and then continuously perfused with an oxygen-saturated bath solution at 2 ml/min. Temperature of the bath solution was set to 35°C and controlled by an inline solution heater (Warner Instruments, Holliston, MA, USA). Whole-cell patch-clamp recordings were performed from the small sized DRG neurons (Cm<40 pF) [22, 23]. Recording electrode resistance after filling it with recording solution was between 4–6 mΩ. Recording solution contained (in mM): K-gluconate (105.0), KCl (30), CaCl_2_ (0.5), MgCl_2_ (2.4), HEPES (10.0), EGTA (5.0), Mg-ATP (5.0), and Na-GTP (0.33). Osmolality was adjusted to 325 mOsm with sucrose with pH adjusted to 7.30 with KOH [20, 21]. Live sensory neurons in the DRG were visualized using a microscope system and approached using a three-dimensional motorized manipulator (Sutter Instrument Company), and whole-cell configurations were established by applying moderate negative pressure after electrode contact and a seal resistance of at least 2 GΩ [20, 21]. Signals of voltage- and current-clamp experiments were amplified via Multiclamp 700B amplifier, filtered at 10 kHz, and sampled at 10 kHz using pCLAMP 10.7 software (Molecular Devices, San Jose, CA, USA) and stored in an IBM computer.

### Western blotting

Mice were deeply anesthetized with 2% isoflurane and laminectomy were performed and dorsal root ganglions between L4-L5 segments were extracted. The dorsal root ganglions were quickly frozen in liquid nitrogen and stored at -80°C for later use. The frozen tissues were homogenized with a hand-held pellet pestle in lysis buffer. Homogenates were then centrifuged at 14000 x g for 20 min at 4°C and the supernatants were collected. The quantification of protein contents was made using the BCA method. Protein samples (20 μg) were electrophoresed in 10% SDS polyacrylamide gels and transferred to polyvinylidene difluoride membrane (Millipore, Bedford, MA, USA). The membranes were blocked with 5% milk and incubated overnight at 4°C with primary antibodies against sodium channel 1.7 (1:500, Cell Signaling), TNFα (1:200, Millipore), IL-1β (1:500, Cell Signaling, Danvers, MA), phospho-ERK (1:1000, Cell Signaling), ERK (1:1000, Cell Signaling), phosphor-AMPK (Thr172) (1:200, Cell Signaling), AMPK (1:200, Cell Signaling), and GAPDH (1:5000, Proteintech, Rosemont, IL). Then the blots were incubated for 1 h at room temperature with a corresponding HRP-conjugated secondary antibody (1:5000; Santa Cruz Biotechnology, CA, USA), visualized in enhanced chemiluminescence (ECL) solution (SuperSignal West Pico Chemiluminescent Substrate, Pierce, Rockford, IL, USA) for 1 min, and exposed onto FluorChem HD2 System. The intensity of immunoreactive bands was quantified using ImageJ 1.46 software (NIH). The ratio of each protein expression over the loading control protein GAPDH was calculated.

### Materials

Compound C was purchased from Tocris (Minneapolis, MN, USA). AICAR was purchased from LC Laboratories (Woburn, MA). All other chemicals were purchased from Sigma (St. Louis, MO, USA).

### Statistical analysis

Using the Kolmogorov-Smirnov Test of Normality, we found that data in the present study does not differ significantly (P > 0.05) from that which is normally distributed. All data are presented as mean ± standard deviation (SD). One- or two-way ANOVA with repeated measures, followed by Bonferroni correction post hoc tests, was used to detect differences in nociceptive behaviors between MRL/Lpr and control mice, and between mice receiving different treatments. The Bonferroni correction, which multiplies the raw P values by the number of comparisons, was used to account for multiple comparisons and obtain the adjusted P values [24, 25]. Whenever applicable, Student’s *t-test* was used to make the comparison between groups (unpaired) or within the same group (paired). Comparisons were conducted as two-tailed tests. A P-value less than 0.05 was considered statistically significant. Statistical analysis was performed using GraphPad Prism 9 (GraphPad Software Inc., San Diego, CA, USA).

## Results

### MRL/Lpr mice develop thermal hyperalgesia at the hind paw

**Fig 1:**
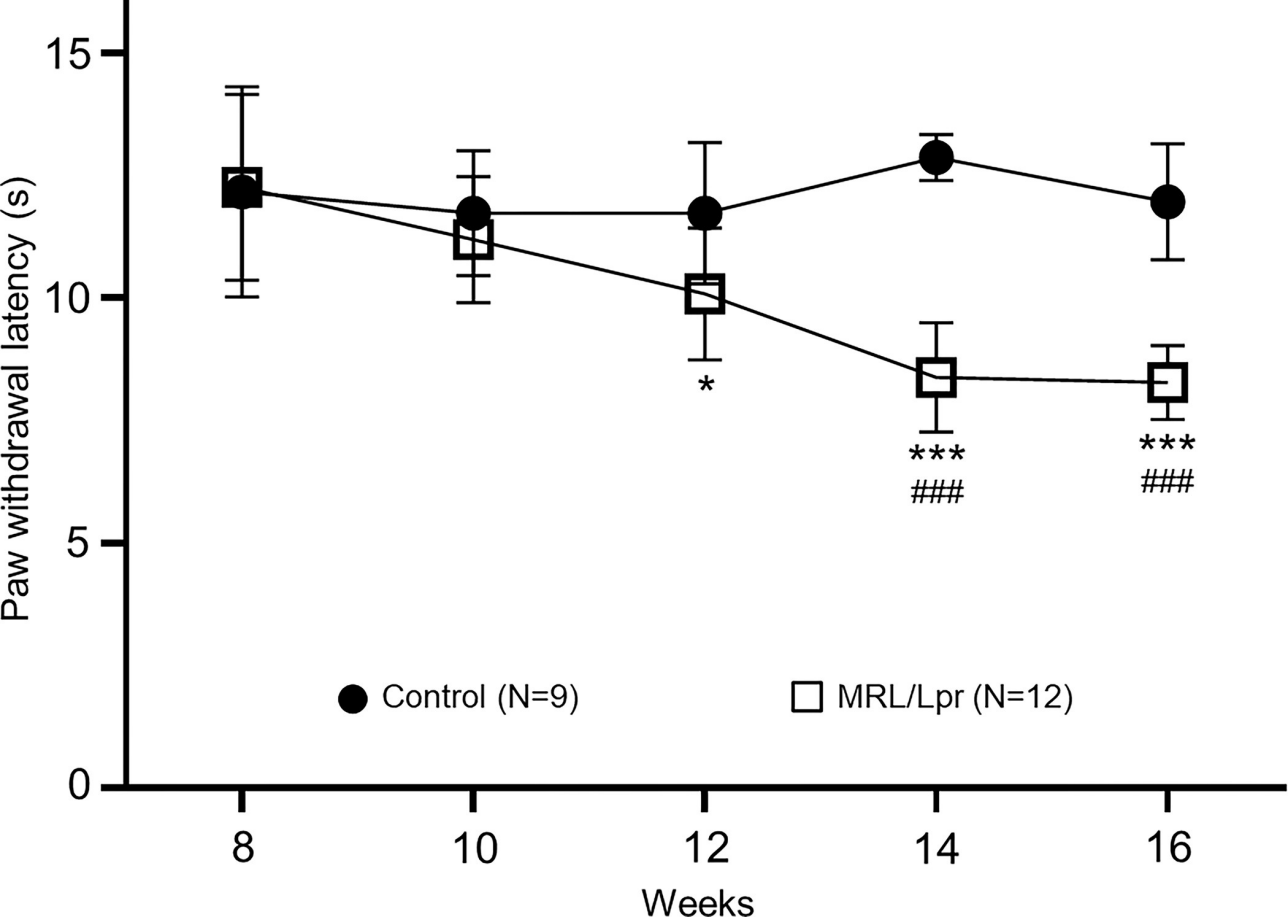
MRL/Lpr mice develop thermal hyperalgesia in the hind paw. Line plots show the average (± SD) of the paw withdrawal latency to radiant heat stimuli between the age of 8 weeks to 16 weeks in MRL/Lpr (N = 12) and control MRL mice (N = 9). Comparisons between data at the age 8 weeks to each of the other weeks in MRL/Lpr mice are labeled with #. Comparisons between MRL/Lpr mice and the control mice at each time point are labeled with *. One symbol: P < 0.05; three symbols: P < 0.001.

To determine whether chronic pain develops in the lupus animal model, latencies of withdrawal response of hind paws to thermal stimuli were measured in control MRL mice (N = 9) and MRL/Lpr (N = 12) mice bi-weekly from the age of 8 weeks. We found that paw withdrawal latencies to thermal stimuli in MRL/Lpr mice (N = 12) and control MRL mice (N = 9) between 8 to 10 weeks of age were similar. At the age of 12 weeks, the paw withdrawal latencies in MRL/Lpr mice (10.09 ± 1.34 s, n = 12) became significantly (P < 0.05) shorter (Fig 1) than those in control MRL mice (11.73 ± 1.45 s, n = 9). This difference became larger with age and reached a plateau between 14 to 16 weeks of age. Meanwhile, paw withdrawal latencies in MRL/Lpr mice (n = 12) between 14 weeks to 16 weeks were significantly (P < 0.001) shorter than their baseline at 8 weeks of age. In contrast, paw withdrawal latency from the control group remained stable from 8 to 16 weeks of age. MRL/Lpr mice during the observation period did not develop any changes in their behaviors or motor functions like grooming or postures. These data indicated that MRL/Lpr mice developed thermal hyperalgesia spontaneously at the age of 12 weeks, which reached a plateau between 14 to 16 weeks. Thus, the data presented below were from control and MRL/Lpr mice at the age of 16 weeks.

### Nociceptive sensory neurons in the DRG from MRL/Lpr mice exhibit hyperexcitability

**Fig 2:**
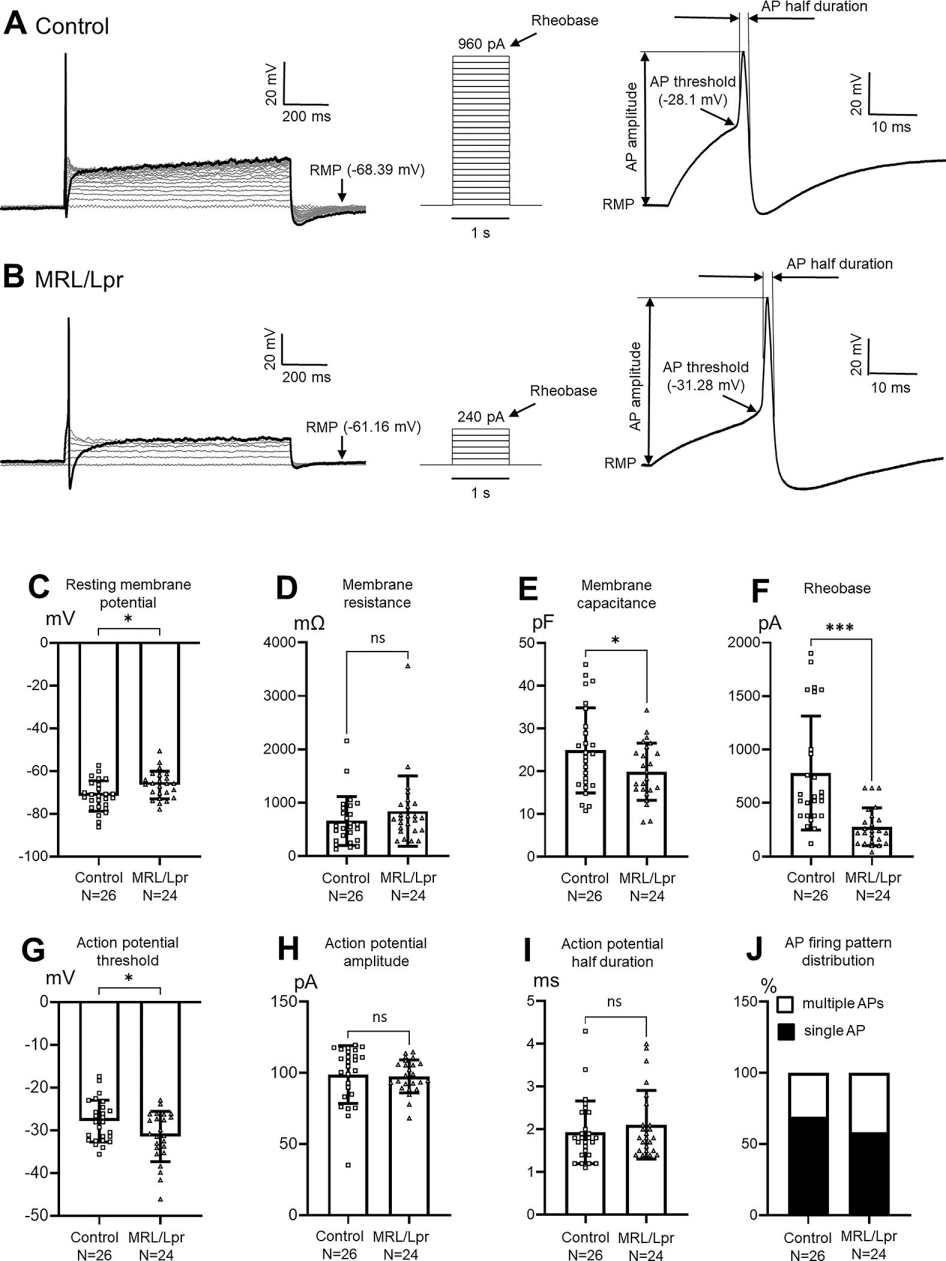
Nociceptive sensory neurons in the DRG from MRL/Lpr mice have increased excitability. Nociceptive sensory neurons in the DRGs from lupus mice have elevation of resting membrane potentials, smaller membrane capacitances, lower rheobases, and more negative action potential thresholds. Raw data (A and B) show samples of membrane potential responses (left) to steps of intracellular current injection (center), and measurements of AP amplitude, AP half duration, and AP thresholds (right) in control **(A)** and lupus **(B)** mice. Note significantly different resting membrane potentials, rheobases, and action potential thresholds between lupus mice and control mice. Bar graphs show the average (+ SD) of resting membrane potential **(C)**, input resistance **(D)**, membrane capacitance **(E)**, rheobase **(F)**, action potential threshold **(G)**, action potential amplitude **(H)**, and action potential half duration **(I)** in nociceptive DRG neurons from control (26 neurons from 15 mice) and lupus mice (24 neurons from 10 mice). **(J)** shows percentage of neurons with multiple APs in the MRL/Lpr group (24 neurons from 10 mice) and the control group (26 neurons from 15 mice). Data obtained from individual neurons are shown in the scatter plot. AP: action potential; RMP: resting membrane potential. * P < 0.05; *** P < 0.001; ns: no significance.

To dissect peripheral mechanisms underlying the genesis of hyperalgesia in MRL/Lpr mice, we compared membrane properties in nociceptive sensory neurons in the DRGs between control MRL mice and MRL/Lpr mice with chronic pain. DRGs were prepared *ex vivo* and DRG neurons were recorded using whole cell configuration. In order to selectively collect nociceptive neurons, only neurons with a small soma (membrane capacitance ≤ 40 pF [22, 23]) and displaying a hump in the repolarization phase (a common feature of primary nociceptive neurons [26, 27]) were used in this study. We first examined whether there are spontaneous action potentials in the sensory neurons under the current clamp mode without current injection. No spontaneous action potentials were found either in 10 MRL/Lpr mice (24 neurons) or in 15 control mice (26 neurons). We then analyzed the passive membrane properties of the neurons. We found that resting membrane potentials (-66.60 ± 1.32 mV, n = 24) in neurons from MRL/Lpr mice (n = 10) were significantly (P < 0.05) more positive than those (-71.73 ± 1.40 mV, n = 26) from the control mice (n = 15) (Fig 2A–2C). Input resistance is a readout for the number of ion channels open (or ion channel conductance) while membrane capacitance reflects the size of the cell and the thickness of the membrane, and affects AP propagation velocity [28]. Input resistances of the recorded neurons were measured with a -10 mV voltage step (1 s duration) from the holding potential (-70 mV) under the voltage clamp mode. We found that input resistances in neurons (n = 24) from MRL/Lpr mice (n = 10) and those (n = 26) from the control mice (n = 15) were similar (Fig 2D) while membrane capacitances (19.93 ± 1.37 pF, N = 24) in neurons from MRL/Lpr mice (n = 10) were significantly (P < 0.05) smaller than those (24.93 ± 1.96 pF, n = 26) in the control mice (n = 15) (Fig 2E). To test the active membrane properties, recordings were made under the current-clamp configuration. Neurons were injected with step currents ranging from 0 to +2000 pA with increments of 20 pA per step (1 s duration), and the membrane potential was recorded. Action potential (AP) amplitude was measured from the resting membrane potential to the peak. AP duration at half-maximal amplitude (AP half duration) was measured as width from 50% of AP upstroke to 50% of repolarization [24]. We found that the minimal currents needed to evoke action potentials (i.e., rheobase) (277.50 ± 32.02 pA, n = 24) from neurons in MRL/Lpr mice (n = 10) were significantly lower (P < 0.001) than those (780.77 ± 104.46 pA, N = 26) in the control mice (n = 15) (Fig 2A, 2B and 2F). Strikingly, action potential (AP) thresholds (-31.47 ± 1.99 mV, n = 24) in MRL/Lpr neurons were significantly (P < 0.05) lower (more negative) than those (-27.87 ± 0.96 mV, n = 26) in the control group (Fig 2A, 2B and 2G). However, we did not find differences between the control and MRL/Lpr groups on AP amplitudes (Fig 2H) or half AP durations (Fig 2I). The elevation of resting membrane potentials, more negative AP thresholds, and the lower rheobase in MRL/Lpr mice neurons all make neurons easier to be activated. These data indicate that MRL/Lpr mice with chronic pain have a significantly higher excitability in nociceptive sensory neurons.

Next, we investigated whether there are differences in action potential firing patterns in the nociceptive DRG neurons between MRL/Lpr mice with chronic pain and control mice. Neurons recorded under the current-clamp mode were injected with a depolarizing current pulse at 2 times rheobase current intensity with 1 s duration. We found that the depolarizing current pulse could elicit a single AP at the initial of the depolarizing current or multiple APs throughout the duration of current injection in both control (15 mice) and MRL/Lpr (10 mice) mice. Among 24 small DRG neurons tested in the MRL/Lpr group, there were 10 neurons displaying multiple APs while the remaining 14 responded with a single AP. We found the percentage of neurons with multiple APs in the MRL/Lpr group (41.67%, 10 of 24) was not significantly different that (30.77%, 8 of 26) in the control group (Fig 2J).

### Cation inward currents, IA, and IK currents are not altered in MRL/Lpr mice

**Fig 3:**
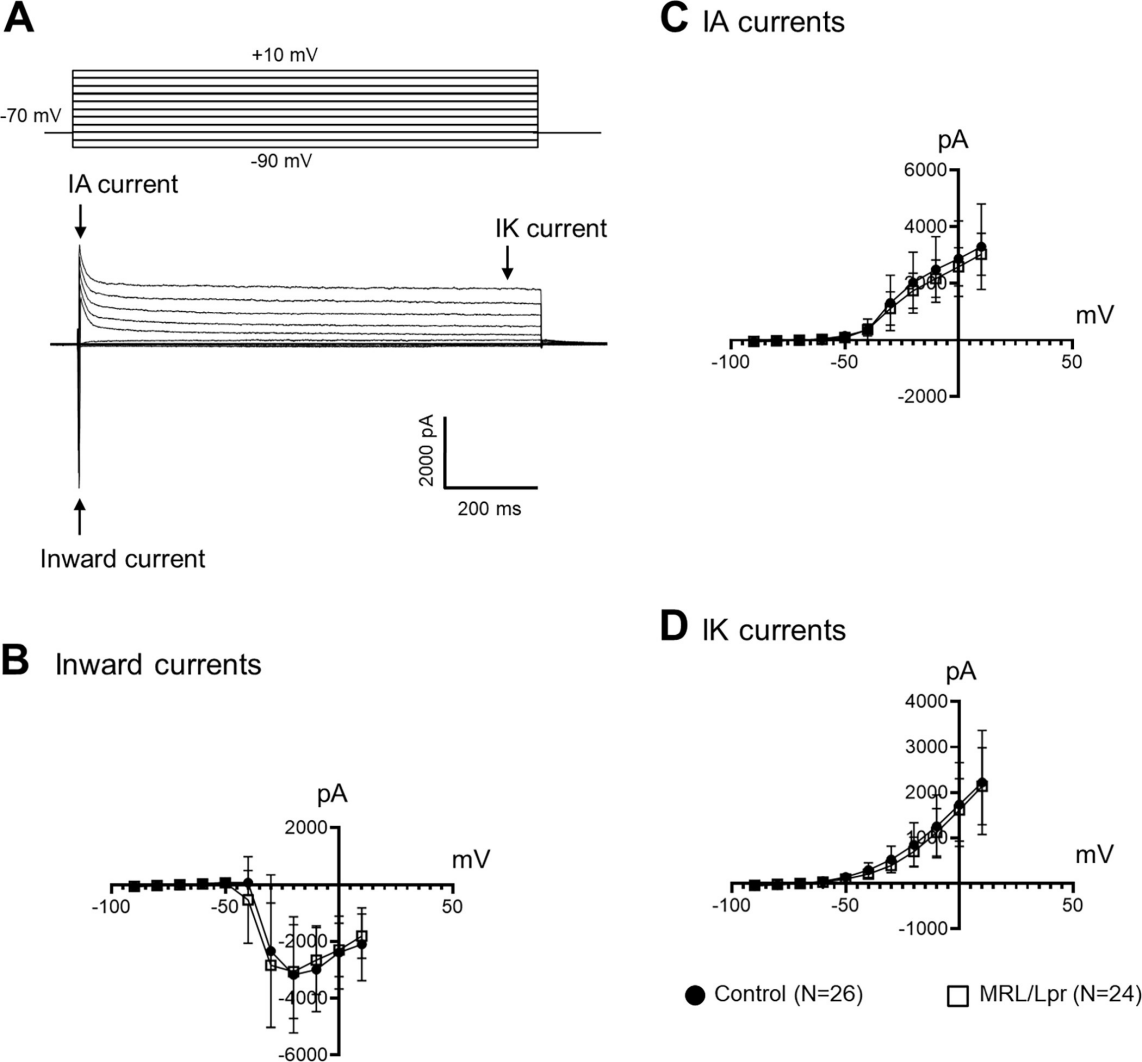
Cation inward currents, IA, and IK currents are not altered in MRL/Lpr mice. **(A):** Raw data show voltage steps of 1s test pulses between -90 mV to +10 mV in 10 mV increments (Top) applied to a neuron, and the current responses from the neuron (Bottom). Inward and outward currents were evoked. The outward current had two components: the inactivating outward component at the initial stage (IA current) and the non-inactivating outward component (IK current). Line plots show summaries (average ± SD) of the inward currents **(B)**, IA currents **(C)**, and IK currents **(D)** obtained from control (26 neurons from 15 mice) and MRL/Lpr mice (24 neurons from 10 mice).

To investigate the ion channel mechanisms underlying changes in membrane properties and neuronal excitability, we examined voltage-activated ion currents in both control (26 neurons from 15 mice) and MRL/Lpr mice (24 neurons from 10 mice). Under voltage-clamp configuration, current-voltage (I–V) relationships were generated by applying a series of voltage steps (from -90 mV to 10 mV in 1 second duration and 10 mV increments at 10 s intervals) from holding potential (-70 mV). The voltage test pulses evoked inward and outward currents in the DRG neurons (Fig 3A). The inward currents are mainly ascribed to the activation of voltage-gated Na^+^ channels while the activation of voltage-dependent K^+^ channels is responsible for the generation of the outward currents [27, 29]. There are two components in the outward components: the inactivating outward component at the initial stage (IA) and the non-inactivating outward component (IK) [27, 30]. We found that there were no significant differences between MRL/Lpr mice (24 neurons from 10 mice) and control mice (26 neurons from 15 mice) in the area under the curve for the I-V plot for the inward currents (Fig 3B), indicating that the cation inward currents in MRL/Lpr mice are similar to those in MRL control mice. These results were consistent with our findings collected under the current-clamp configuration that AP amplitudes between MRL/Lpr mice and control mice were not significantly different. Upon analysis of the outward currents, we found that the IA and IK currents recorded from nociceptive DRG neurons (N = 24) in MRL/Lpr mice (10 mice) were similar to their counterparts (N = 26) in control mice (15 mice) (Fig 3C and 3D). We further analyzed the current densities of each current for each cell by dividing its peak currents with cell capacitance. We found that current densities for inward currents, and both IA and IK outward currents in MRL/Lpr mice were similar to those in control mice (S1 Data).

### MRL/Lpr mice have no changes in Nav1.7 protein expression but increased protein expression of TNAα, IL-1β, phosphorylated ERK1/2, and suppression of AMPA activity in the DRGs

**Fig 4:**
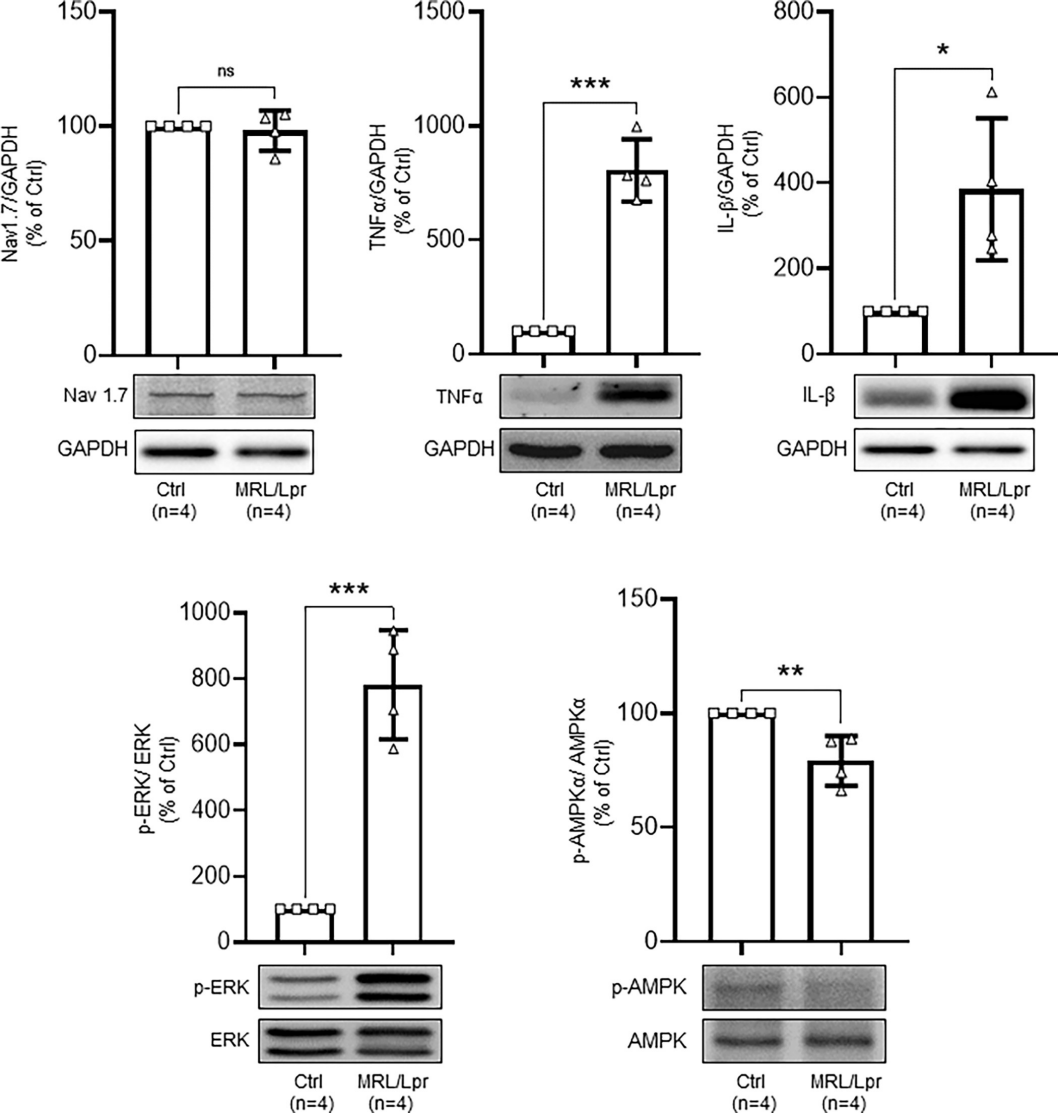
MRL/Lpr mice have no changes in Nav1.7 protein expression but increased protein expression of TNAα, IL-1β, phosphorylated ERK1/2, and suppression of AMPA activity in the DRGs. Bar graphs show protein expression (mean + SD) ratios of Nav1.7, TNFα, IL-1β to GAPDH, the ratio of phosphorylated ERK (pERK) to ERK, the ratio of phosphorylated AMPK to total AMPK in the DRGs from four MRL/Lpr mice and four control MRL mice. Data obtained from individual animals are shown in the scatter plot. Samples of each protein molecule expression in each group are shown below. * P < 0.05; ** P < 0.01; *** P < 0.001.

Given sodium channel 1.7 (Nav1.7) is a major voltage-gated sodium channel for sensory neurons in the DRGs, we next determined the protein expression of Nav1.7 in the DRGs between L4 to L5 spinal segments. We found no significant difference in the protein expression of Nav1.7 in the DRGs between MRL/Lpr mice (4 mice) with thermal hyperalgesia and MRL control mice (4 mice) (Fig 4). These findings were consistent with our patch clamp results that the peak inward currents (Fig 3B) and AP amplitudes (Fig 2H) in MRL/Lpr mice were similar to those in control mice. Previous studies demonstrated that over-production of pro-inflammatory cytokines in the DRG leads to increases in nociceptive sensory neuronal excitability, and activity of extracellular signal-regulated kinase (ERK; a marker for neuronal hyperactivation). We then examined levels of TNAα and IL-1β, as well as phosphorylated ERK (pERK) in the DRGs obtained from control and MRL/Lpr mice with chronic pain at the age of 16 weeks. We found that protein expression levels of TNFα, IL-1β, and pERK were significantly (P < 0.05) elevated in MRL/Lpr mice in comparison with those in MRL control mice (Fig 4). Given that AMPK negatively regulates pERK activity in DRG neurons [31] and other cells [32], levels of phosphorylated AMPK in the DRG were analyzed by western blotting. As shown in Fig 4, the protein expression level of phosphorylated AMPK was significantly (P < 0.01) suppressed in the DRG of MRL/Lpr mice in comparison with that in control mice. These data indicate that AMPK activity in the DRG is suppressed in MRL/Lpr mice with thermal hyperalgesia.

### Suppression of the AMPK activity leads to thermal hyperalgesia

**Fig 5:**
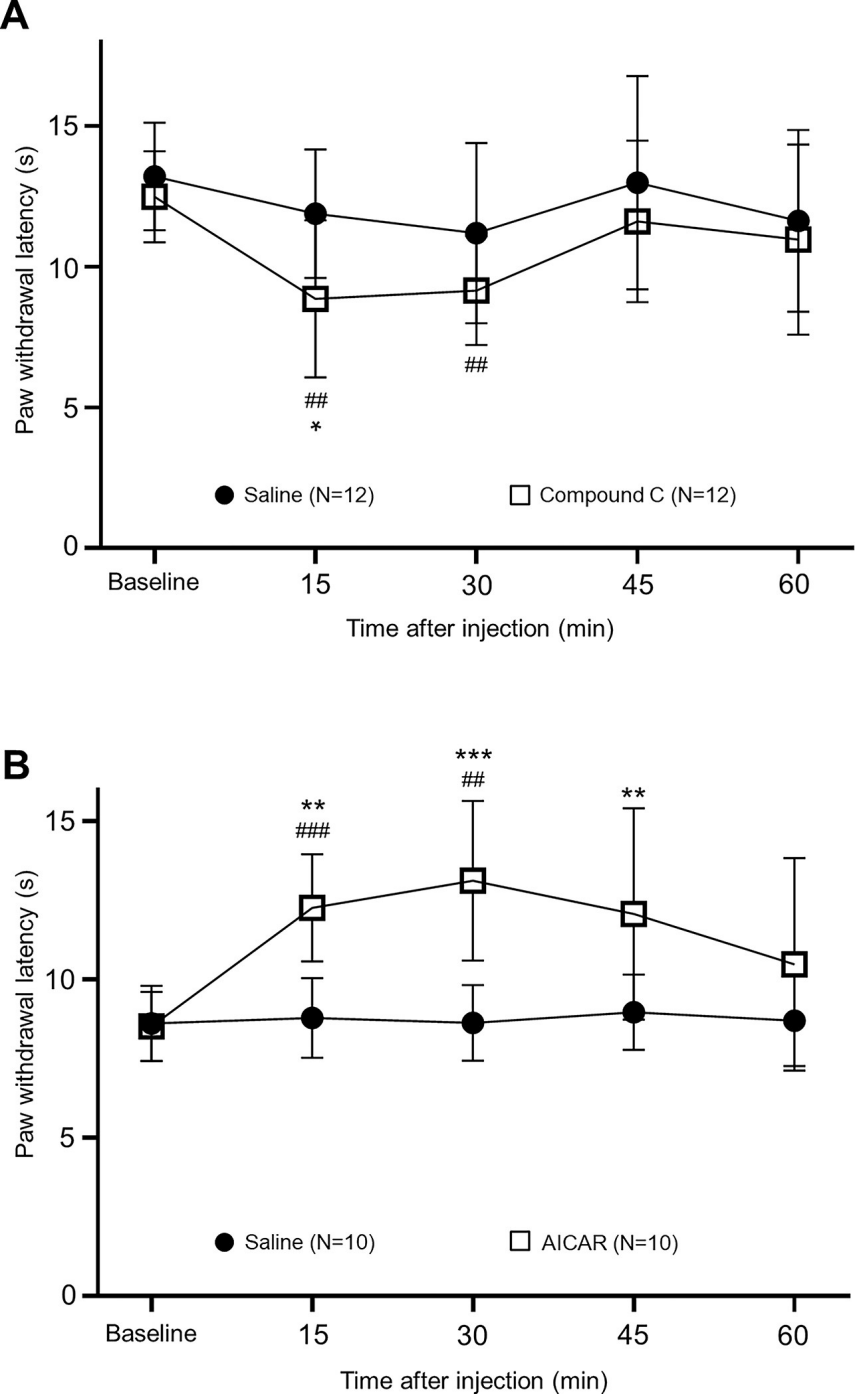
Suppression of the AMPK activity leads to thermal hyperalgesia while activation of AMPK reduces thermal hyperalgesia. **(A):** Line plots show summaries (means ± SD) of the paw withdrawal latency to radiant heat stimuli in control mice immediately before (baseline) and after intraplantar injection of Compound C (concentration: 25 μMol; volume: 25 μl; N = 12) or saline (25 μl, N = 12) was made. **(B):** Line plots show summaries (means ± SD) of the paw withdrawal latency to radiant heat stimuli in MRL/Lpr mice immediately before (baseline) and after intraplantar injection of AICAR (concentration: 1 mMol, volume: 25 μl; N = 10) or saline (25 μl, N = 10). Comparisons between data before and different time points after injection of compound C (A) or AICAR (B) are labeled with #. Comparisons of saline treated group and Compound C treated group (A) or AICAR treated group (B) are labeled with *. One symbol: P < 0.05; Two symbols: P < 0.01; Three symbols: P < 0.001.

Given that AMPK is reportedly expressed in the peripheral sensory neurons [33, 34], we speculated that the suppressed AMPK activity in sensory neurons contributes to the genesis of thermal hyperalgesia in lupus mice. Thus, we determined if thermal sensitivity in the hind paw is altered in normal control mice when an AMPK inhibitor is subcutaneously injected. Compound C, a known selective and reversible AMPK inhibitor [35], was used. After measuring baseline latencies of withdrawal response to radiant heat stimuli, intraplantar injection of Compound C (concentration: 25 μMol; volume: 25μl) was made in the left hind paw. As shown in Fig 5A, topical subcutaneous injection of Compound C but not vehicle treatment (saline, 25μl) induced thermal hyperalgesia in the normal control mice. In comparison with their baseline measurements (12.49 ± 1.62 s, n = 12), the withdrawal response latencies to heat stimuli in the normal control mice (n = 12) receiving Compound C were significantly decreased to 8.86 ± 2.80 s (n = 12, P < 0.01) at 15 min and 9.15 ± 1.93 s (n = 12, P < 0.01) at 30 min following drug treatment. Meanwhile, saline treatment did not significantly change withdrawal response latencies to heat stimuli in the animals (n = 12). Moreover, in comparison with normal control mice (n = 12) treated with vehicle (saline, 25μl), withdrawal response latencies to heat stimuli in the normal control mice (n = 12) receiving Compound C were also significantly shorter at 15 min (n = 12, P < 0.05).

Next, we determined whether thermal hyperalgesia in MRL/Lpr mice can be ameliorated by a widely used AMPK activator, 5-amino-4-imidazole carboxamide (AICAR) [36]. We found that subcutaneous intraplantar injection of AICAR (concentration: 1 mMol, volume: 25 μl) significantly increased the hind paw withdrawal latencies to radiant heat stimuli from 8.52 ± 1.09 s (n = 10) at baseline to 12.26 ± 1.69 s at 15 min (n = 10, P < 0.001), and 13.12 ± 2.52 s (n = 10, P < 0.01) at 30 min (Fig 5B). The hind paw withdrawal latencies in MRL/Lpr mice (n = 10) treated with vehicle (saline, volume: 25μl) remained unchanged. Furthermore, in comparison with MRL/Lpr mice (n = 10) treated with vehicle (saline, 25μl), withdrawal response latencies to heat stimuli in MRL/Lpr mice (n = 10) treated with AICAR were also significantly increased at 15 min (n = 10, P < 0.01), 30 min (n = 10, P < 0.001) and 45 min (n = 10, P < 0.01) (Fig 5B). Together with the results in Fig 5A, these findings suggest that suppression of AMPK activity in the sensory neurons contributes to the genesis of thermal hyperalgesia in MRL/Lpr mice.

### Pharmacological suppression of AMPK activity in normal nociceptive DRG neurons recapitulates membrane properties found in MRL/Lpr mice with thermal hyperalgesia

**Fig 6:**
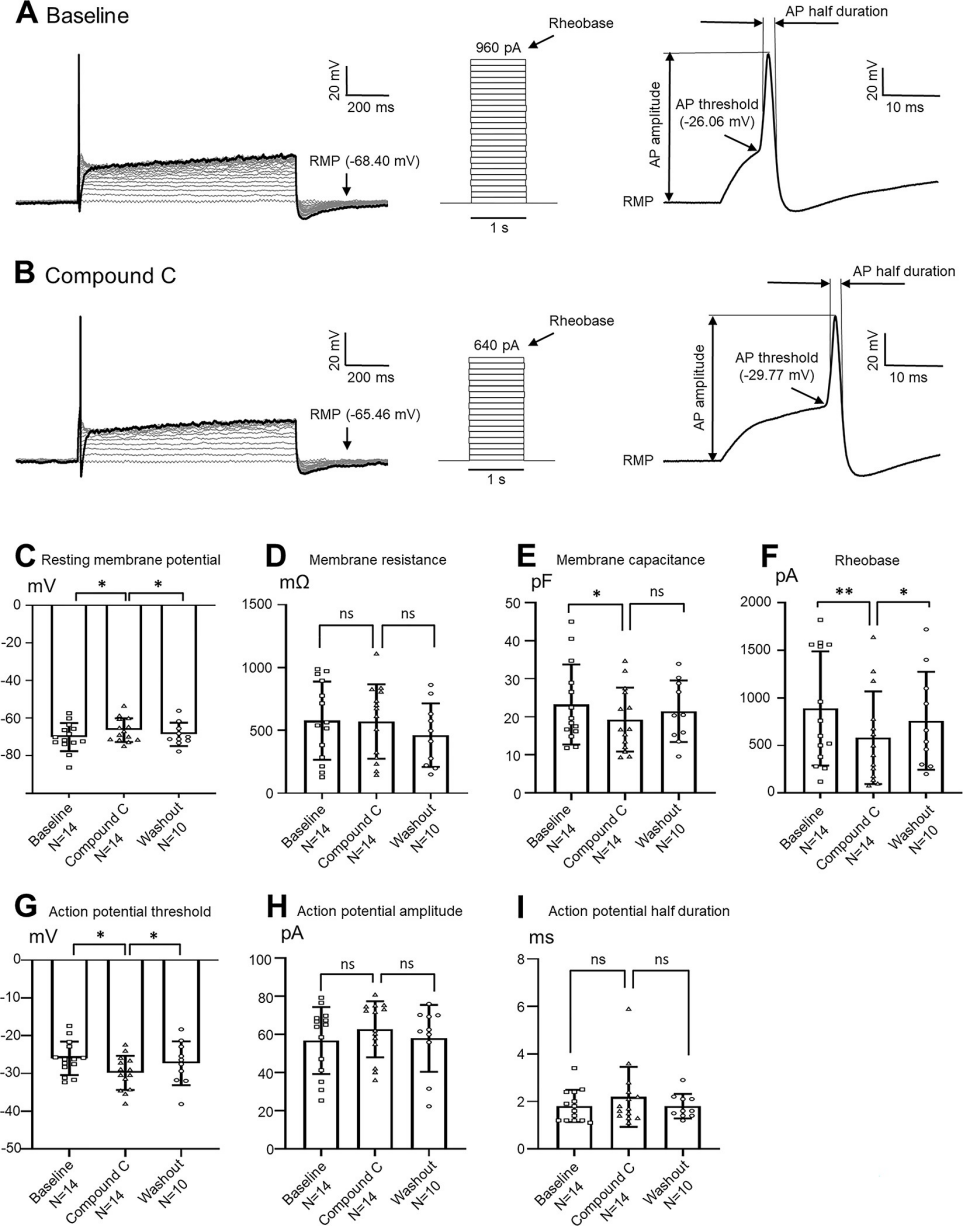
Pharmacological suppression of AMPK activity in normal nociceptive DRG neurons recapitulates membrane properties found in MRL/Lpr mice with thermal hyperalgesia. Raw data (**A** and **B**) show samples of membrane potential responses (left) to steps of intracellular current injection (middle), and measurements of AP amplitude, AP half duration, and AP thresholds (right) before (baseline) **(A)** and during perfusion of compound C (concentration in the bath: 10 μMol) **(B)**. Note different resting membrane potentials, rheobases, and action potential thresholds between baseline, and during perfusion of compound C. Bar graphs show the average (+ SD) of resting membrane potential **(C)**, input resistance **(D)**, membrane capacitance **(E)**, rheobase **(F)**, action potential threshold **(G)**, action potential amplitude **(H)**, and action potential half duration **(I)** in 14 nociceptive DRG neurons from 9 control mice before (baseline), during perfusion, and washout of compound C. Data obtained from individual neurons are shown in the scatter plot. AP: action potential; RMP: resting membrane potential. * P < 0.05; ** P < 0.01; ns: no significance.

Finally, we postulated that deficient AMPK activity in nociceptive DRG neurons in MRL/Lpr mice is a culprit causing hyperexcitability in the nociceptive DRG neurons and thermal hyperalgesia in lupus mice. To test this hypothesis, we directly examined the effects of Compound C on nociceptive DRG neurons obtained from nine normal control mice using whole cell patch clamp techniques. After recording neuronal baseline activity, we suppressed AMPK in the recorded neurons via bath perfusing Compound C (concentration in the bath: 10 μMol) [14]. We first observed whether suppression of AMPK induces spontaneous action potentials in the nociceptive DRG neurons. In 14 neurons tested, no spontaneous action potentials were induced. Importantly, perfusion of Compound C significantly (P < 0.05) raised the resting membrane potential from -70.07 ± 2.00 mV to -66.38 ± 1.68 mV (n = 14) (Fig 6A–6C), and significantly (P < 0.01) reduced the membrane capacitance from 23.24 ± 2.81 pF to 19.27 ± 2.25 pF (n = 14) (Fig 6E) without changes in the input resistance (Fig 6D) in the nociceptive DRG neurons. We then assessed the effects of Compound C on the active membrane properties in the nociceptive DRG neurons. Suppression of AMPK activity with bath perfusion of Compound C significantly (P < 0.01) reduced the rheobase from 888.60 ± 160.30 pA to 581.40 ± 130.10 pA (n = 14) (Fig 6F), and significantly (P < 0.05) reduced the AP threshold from -25.99 ± 1.18 mV to -29.86 ± 1.12 mV (n = 14) (Fig 6A and 6G), but did not significantly alter the AP amplitude (Fig 6A and 6H) and the AP duration (n = 14) (Fig 6A and 6I). The effects induced by Compound C dissipated after washout of Compound C. Furthermore, we also examined whether suppression of AMPK activity in the nociceptive DRG neurons in control mice can alter AP firing patterns. Fourteen neurons (11 neurons with single APs and 3 neurons with multiple APs) were tested. After perfusion of Compound C (concentration in the bath: 10 μMol), only one neuron displayed a change of AP firing patterns from a single AP pattern to a multiple AP pattern. AP firing patterns in the other 13 neurons remained unchanged in the presence of Compound C. Taken together with the behavioral data shown in Fig 5, our data indicate that deficient AMPK activity in the nociceptive DRG neurons results in enhanced excitability in the small DRG neurons and chronic pain in MRL/Lpr mice.

## Discussion

Our present study reveals peripheral mechanisms underlying the genesis of chronic pain in mice induced by SLE. We found that excitability in nociceptive sensory neurons in the DRGs of MRL/Lpr mice with thermal hyperalgesia is significantly enhanced, and suppression of AMPK activity is a culprit causing hyperexcitability in the nociceptive sensory neurons and thermal hyperalgesia induced by SLE. Our findings suggest that aberrant membrane properties and suppression of AMPK in nociceptive sensory neurons are critical mechanisms underlying peripheral sensitization induced by lupus disease.

Sensory neurons have been considered as the first gate controlling nociceptive signaling. This gate is orchestrated by processes related to sensory transduction, as well as AP initiation and propagation in sensory neurons. Altered passive and active membrane properties in nociceptive sensory neurons are culprits leading to pathologic AP initiation and propagation in animals with chronic pain. Little is known about the membrane properties in nociceptive sensory neurons of mice with chronic pain caused by lupus. Our present study found that resting membrane potentials were significantly elevated in nociceptive sensory neurons of mice with chronic pain caused by lupus. Similar findings were reported in animals with neuropathic pain induced by nerve ligation [29, 37], spinal cord injury [38], diabetes [39], and pain induced by arthritis [40], or bone cancer [10], but not in animals with inflammatory pain induced by CFA [9]. Interestingly, while nociceptive sensory neurons in lupus mice displayed similar membrane resistances as control mice, their capacitances were significantly smaller. These contrast with previous findings from animals with inflammatory pain induced by CFA, where capacitances were not altered [9]. Nociceptive DRG neurons in rats with neuropathic pain [37, 41] and chronic pain caused by arthritis [40] or bone cancer [10] have decreased action potential thresholds. Rheobases in small nociceptive sensory neurons are reduced in animals with nerve injury [7, 8, 37] and inflammatory pain [9]. We extended these observations by finding a reduction of AP thresholds and rheobases in nociceptive sensory neurons of mice with chronic pain caused by lupus. The elevated resting membrane potentials, smaller membrane capacitance, reduced AP thresholds, and rheobases in nociceptive neurons in lupus mice all enhance the responsiveness of these neurons to low-threshold stimuli in vivo, ultimately resulting in enhanced pain perception.

Although AP durations and amplitudes were reportedly increased in nociceptive sensory neurons from animals with nerve injury [37] and inflammatory pain induced by CFA [9], we did not find significantly altered AP durations and amplitudes in lupus mice with chronic pain. Furthermore, no spontaneous APs were found in nociceptive sensory neurons either from normal controls or mice with chronic pain caused by lupus. AP firing pattern evoked by current injection in nociceptive sensory neurons of lupus mice with chronic pain and those of control mice were similar. These findings deviate from previous reports about nociceptive neurons from animals with neuropathic pain induced by chronic constriction of the sciatic nerve [42] and animals with bone cancer pain [10], where a significantly increased incidence of spontaneous AP activities was found. The unique membrane properties in lupus mice with chronic pain suggest a different mechanism underlying peripheral sensitization caused by lupus disease.

Multiple signaling molecules are implicated in the regulation of resting membrane potentials in nociceptive sensory neurons. Resting membrane potentials in nociceptive sensory neurons are depolarized by pro-inflammatory mediators, like nerve growth factor [40], serotonin [43], proteases [44], and osteoarthritic synovial fluid [45]. In animals with spinal cord injury, it was reported that elevation of resting membrane potentials in nociceptive sensory neurons is maintained by cAMP via PKA [38]. Activation of ERK was also suggested to cause depolarization of resting membrane potentials [46]. In this study, we found that resting membrane potentials in nociceptive DRG neurons in lupus mice with thermal hyperalgesia were elevated in comparison with normal controls. Furthermore, lupus mice with chronic pain had low AMPK activity in the DRG. When nociceptive normal DRG neurons were treated by an AMPK inhibitor, Compound C, resting membrane potentials were rapidly depolarized (within less than 6 min after drug application). The rapid action induced by the suppression of AMPK activity suggests that such changes are not ascribed to de novo protein synthesis.

Previous studies have shown that resting membrane potentials in nociceptive DRG neurons are determined by conductance and activities in K2P “leak” channels, M channels (Kv7, KCNQ), 4-AP-sensitive K_V_ (including Kv1.4, Kv2s, and Kv3.4) channels, as well as Na^+^–K^+^ ATPase pump [47]. Reduced conductance in these channels would raise the membrane resistance and reduce outward cationic current, resulting in the elevation of resting membrane potential. For example, it was reported that in animals with neuropathic pain, TREK2 function (a major subtype of K2P channels) in the cell membrane in nociceptors is reduced. This causes a reduction of conductance in K2P “leak” channels and elevation of resting membrane potentials, which contributes to increased DRG excitability after nerve injury [48, 49]. Our present study found that the elevation of resting membrane potentials in nociceptive DRG neurons in lupus mice, and in normal mice upon inhibition of AMPK activity, was not associated with significant changes in membrane resistance. Thus, it is unlikely the elevated resting membrane potentials found in our study are caused by a reduced conductance in the membrane channels to outward cationic current. Rather, the altered function of Na^+^–K^+^ ATPase pump may be involved. Previous studies demonstrated that suppression of Na^+^–K^+^ ATPase activity contributes to increased excitability in neurons [50] while enhanced Na^+^–K^+^ ATPase activity leads to hyperpolarization of resting membrane potential without changes in membrane resistance [51]. Interestingly, studies from multiple cell lines have shown that AMPK is the Na^+^–K^+^ ATPase activator [52, 53]. These findings are in line with our finding that inhibition of AMPK resulted in depolarization in the resting membrane potential in nociceptive DRG neurons.

Another salient feature in nociceptive DRG neurons of lupus mice is a reduction of AP activation thresholds without a significant alteration in AP amplitudes. Changes in several ion channels knowingly contribute to the reduction of the AP threshold. Voltage-gated A-type K^+^ currents (IA currents) are considered to function as a brake to counteract membrane depolarization, and thereby modulate the AP action threshold and shape in sensory neurons [54]. In the present study, IA currents in lupus mice with chronic pain and control mice were similar (Fig 3), suggesting that IA currents is not involved in the reduction of AP thresholds found in lupus mice. Nav1.7 channel is a key threshold channel for the activation of nociceptive DRG neurons [55]. It was shown that ERK phosphorylates sodium channel Nav1.7, leading to reduced activation thresholds of the voltage-dependent Nav1.7 channels [46]. Activation of ERK is known to be a key convergent transducer activated by many pro-inflammatory cytokines and implicated in hyperexcitability in sensory neurons induced by nerve injury and inflammation [56, 57]. Consistent with these previous findings, we found that levels of phosphorylated ERK and TNFα and IL-1β protein in lupus mice were higher than those in normal controls. Furthermore, it has been shown that activation of AMPK suppresses ERK activation [13, 58] while suppression of AMPK with Compound C leads to phosphorylation of ERK [46]. Therefore, it is conceivable that suppression of AMPK activity in the sensory neurons in lupus mice results in activation of ERK, which in turn causes phosphorylates sodium channel Nav1.7, and lowers the AP activation threshold in nociceptive sensory neurons. It would be interesting for future studies to further dissect ionic mechanisms underpinning the changes in membrane properties in lupus mice with chronic pain.

Mechanisms underlying chronic pain caused by lupus disease has been mainly investigated in female MRL/Lpr mice. It was reported that female MRL/Lpr mice with chronic pain exhibit increased excitatory synaptic activity and suppressed activity of glutamate transporters in the spinal dorsal horn [3, 4]. Such pathological changes were attributed to increased expression of IL-1β, IL-18 [3, 4], thrombin, and suppression of AMPK activity [59] in the spinal dorsal horn. Our present study extended the role of AMPK in chronic pain to the peripheral nociceptive DRG neurons in male MRL/Lpr mice. We found that suppression of AMPK activity results in elevation of resting membrane potentials, reduction of AP activation thresholds, and rheobases in nociceptive DRG neurons, which ultimately leads to enhanced nociceptive neuronal excitability and contributes to the genesis of chronic pain induced by lupus disease. Given that suppression of AMPK activity in nociceptive DRG neurons is implicated in the development of pathological pain induced by incision [60] and lumbar disk puncture [61] in both male and female rodents, it is conceivable that AMPK in nociceptive DRG neurons could also plays an important role in the development of chronic pain caused by lupus disease in females. Further studies are warranted to validate this notion.

In conclusion, our study demonstrated that the hyperexcitability in peripheral nociceptive sensory neurons contributes to the genesis of thermal hyperalgesia in mice with SLE, and suppression of AMPK activity in the nociceptive neurons is a culprit causing elevation of resting membrane potentials, lowering of AP thresholds and rheobases in nociceptive sensory neurons and thermal hyperalgesia. Our study provides a basis for targeting signaling pathways regulating membrane properties of nociceptive DRG neurons as a means for conquering chronic pain caused by SLE.

## Supporting information

S1 Data (XLSX)

S1 Fig (XLSX)

S2 Fig (XLSX)

S3 Fig (XLSX)

S4 Fig (XLSX)

S5 Fig (XLSX)

S6 Fig (XLSX)
